# SETDB1 promotes the progression of colorectal cancer via epigenetically silencing p21 expression

**DOI:** 10.1038/s41419-020-2561-6

**Published:** 2020-05-11

**Authors:** Nan Cao, Yali Yu, Hua Zhu, Meng Chen, Ping Chen, Mingxing Zhuo, Yujuan Mao, Lianyun Li, Qiu Zhao, Min Wu, Mei Ye

**Affiliations:** 1 Department of Gastroenterology, Zhongnan Hospital of Wuhan University, Wuhan, Hubei 430071 China; 2 Hubei Clinical Center and Key Lab of Intestinal and Colorectal Diseases, Wuhan, Hubei 430071 China; 3 College of Life Sciences, Wuhan University, Wuhan, Hubei 430072 China

**Keywords:** Cell proliferation, Cancer genetics

## Abstract

SETDB1, a histone H3K9 methyltransferase, has been reported to be upregulated in a variety of tumors and promotes cancer development. However, the exact pathogenesis of SETDB1 in human colorectal cancer (CRC) is hitherto unknown. Here, we showed that SETDB1 expression was highly amplified in CRC. Functionally, SETDB1 downregulation in SW480 and HCT116 cells reduced cell proliferation, migration, invasion, and increased CRC cells apoptosis. In contrast, SETDB1 overexpression promoted CRC cells proliferation, migration, and invasion. High expression of SETDB1 was associated with a more aggressive phenotype in vitro. Flow cytometry showed that cell cycle was arrested in G1 phase after SETDB1 silencing. Furthermore, depletion of SETDB1 in vivo suppressed CRC cells proliferation. Mechanistically, p21 was identified as the target of SETDB1. After transfected with siSETDB1, expression of p21 was distinctly increased. In contrast, expression of p21 was significantly decreased after overexpression SETDB1. We also showed that SETDB1 could be involved in the regulation of epithelial–mesenchymal transition (EMT) in HCT116 cells. Moreover, we confirmed that SETDB1 could regulate the activity of p21 promoter by dual-luciferase repoter assay, and proved that SETDB1 could bind to the promoter of p21 and regulate its H3K9me3 enrichment level by ChIP-PCR experiment. Finally, we verified that silencing of SETDB1 inhibited CRC tumorigenesis in vivo. In conclusion, our results indicate that SETDB1 is a major driver of CRC development and might provide a new therapeutic target for the clinical treatment of CRC.

## Introduction

Colorectal cancer (CRC) is the third leading cause of cancer death in men and the second leading cause of cancer death in women, according to the latest global cancer statistic^1^. It is well known that CRC carcinogenesis is complicated and still has not been defined, involving multiple genomic variations and biological processes. Therefore, the molecular mechanism must be further studied to deepen the understanding of CRC.

Epigenetics plays a central role in the pathogenesis of cancer^2^. Epigenetics changes are independent of DNA sequence, mainly including DNA methylation and histone modification, which are reversible features^3,4^. Remarkably, histone can be modified at many sites^5^. Recent evidence indicates that histone methyltransferases (HMTs) and histone acetyltransferase such as EZH2, JMJD3, UTX, SMYD3, and KAT2A are dysregulated in human cancers^6–8^. Previous study showed that p300, a histone acetylase, cooperates with CREPT in CRC activating Wnt/β-catenin signaling pathway, thus promoting the development of CRC^9^. Increasing the level of H3K27me3 can improve drug sensitivity of CRC patients^10^. In addition, histone deacetylase (HDAC) inhibitors against CRC are already available in clinical^11,12^. Moreover, a recent study in vivo showed that the offsprings of KDM6A conditional knockout male mice exhibited increased incidence of tumors^13^. These studies suggest that histone modifications play important roles during the occurrence of human cancer. Cancer genome sequencing evidenced that heterochromatin-associated histone modification H3K9me3 accounts for more than 40% of mutation-rate variation^14^, and more recently study exhibited the increasing expression of H3K9me3 is associated with treatment-resistant phenotypes^15^. These suggest that H3K9me3 and its enzymes may be potential therapeutic targets.

SET domain bifurcated 1 (SETDB1) is also known as ESET/KMT1E and located on human chromosome 1q21^16^. SETDB1 is characterized by histone H3K9 methyltransferase activity^17^, which can catalyze the trimethylation of H3K9^18,19^. H3K9me3 represents a specific tag for epigenetic repression by recruiting heterochromatin protein1 to methylated histones^20^. Dysregulation of SETDB1 can affect the normal development of embryos and participate in the pathogenesis of tumors^21,22^. Recent years, increasing evidence showed that abnormal expression of SETDB1 is closely related to tumorigenesis, including melanoma, lung cancer, breast cancer, and liver cancer^23–26^, suggesting that SETDB1 is involved in the pathogenesis of a variety of human cancers. Moreover, recent literatures have reported that SETDB1 expression is associated with poor clinical outcomes in CRC and is thought to be a dysregulated epigenetic regulator^27–29^. However, the role of SETDB1 in CRC is still controversial and has not been defined, the exact pathogenesis needs further investigations.

p21, also known as CDKN1A and a member of the Cip/Kip family, is a key regulator of cell cycle and cell senescence^30,31^. A recent study reveals that mesenchymal stem cells help CRC cells resist senescence through p53/p21 pathway^32^. In addition, histone modification has an important role in regulating p21 expression. EZH2 promotes tumor progression in melanoma cells through epigenetically silencing p21 expression^33^. 4-Nonylphenol could interact with RNF2 and EZH2 resulting in decreased p21 expression via increasing H3K27me3 repressive marks at p21 promoter and promoting MCF-7 cells proliferation^34^. In multiple myeloma (MM), hydroxamicacid-based small-molecule *N*-hydroxy-4-(2-[(2-hydroxyethyl) (phenyl) amino]-2-oxoethyl) benzamide (HPOB), a novel HDAC inhibitor, could induce MM cells death via transcriptional activation of p21^35^. Furthermore, lncRNA CRNDE binding with EZH2 promotes the proliferation of CRC cells by epigenetic silencing of DUSP5/p21 expression^36^, and another lncRNA HOXA-AS2 can inhibit the expression of p21 and KLF2 in CRC cells by binding with EZH2 and LSD1, which both regulate histone methylation^37^.

Given the great importance of epigenetic regulators in CRC, in the current study, we investigated the oncogenicity of SETDB1 in CRC and shed light on the importance of SETDB1 in the proliferation, cell cycle, apoptosis, migration, and invasion of CRC cells. We defined SETDB1 as an oncogene via elucidating the epigenetic regulation of the p21 expression. In addition, alteration of epithelial–mesenchymal transition (EMT) markers was observed after inhibition of SETDB1 in HCT116 cells. These results suggest that SETDB1 has the potential to serve as a novel biomarker and therapeutic target in CRC.

## Results

### SETDB1 is upregulated in CRC tissues

**Fig. 1:**
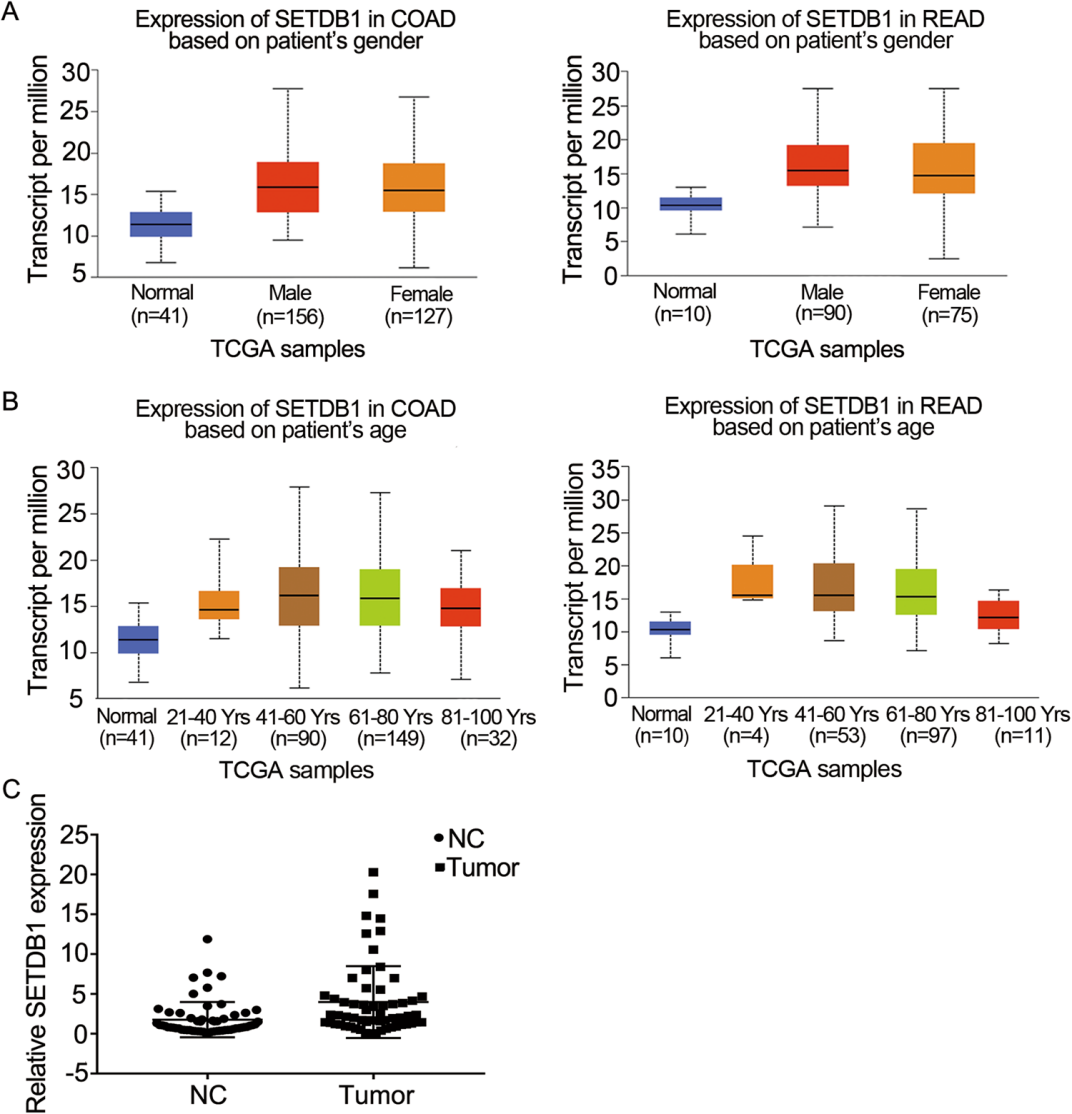
SETDB1 expression in CRC tissues. **a**, **b** Higher SETDB1 expression in COAD and READ tumor tissues of different genders and ages according to UALCAN analysis. **c** Relative expression of SETDB1 in 60 pairs CRC tissues. *P* = 0.0090, *P* < 0.05.

We applied UALCAN database^38^ to analyze the expression of SETDB1 in different genders and ages of colon adenocarcinoma (COAD) and rectum adenocarcinoma (READ), and the result showed that SETDB1 was upregulated in both colon and rectal cancer regardless of genders and ages (Fig. 1a, b). To further confirm the expression of SETDB1 in CRC, we examined the expression of SETDB1 in tumor and adjacent normal tissues of 60 CRC patients, the result of qRT-PCR showed that SETDB1 was highly expressed in cancer tissues compared to the controls (Fig. 1c). However, the expression of SETDB1 was not related to the clinical characteristic of CRC, including age, gender, location, pathological differentiation, depth of tumor, lymph node metastasis, distant metastasis, and tumor stage (Table 1). The results indicate that SETDB1 expression is associated with the development of CRC.

**Table 1:** Correlation between SETDB1 expression and clinicopathologic variables in patients with CRC (*n* = 60).

Characteristics	Number	Percent	SETDB1		Chi-square test
	(*n* = 60)		High	Low	*P* value
Ages (years)					0.481
<60	16	27%	14	2	
≥60	44	73%	33	11	
Gender					0.609
Male	36	60%	29	7	
Female	24	40%	18	6	
Tumor location					0.561
Right colon	13	21.7%	9	4	
Left colon	22	36.7%	17	5	
Rectum	25	41.6%	21	4	
Pathological differentiation					0.578
Poor	13	21.7%	10	3	
Well and moderate	47	78.3%	37	10	
Depth of tumor					0.673
T1 and T2	8	13%	7	1	
T3 and T4	52	87%	40	12	
Lymph node metastasis					0.330
N0	35	58.4%	26	9	
N1	17	28.3%	13	4	
N2	8	13.3%	8	0	
Distant metastasis					0.783
M0	59	98.3%	46	13	
M1	1	1.7%	1	0	
Tumor stage					0.368
I and II	35	58%	26	9	
III and IV	25	42%	21	4	

### SETDB1 promotes CRC cell proliferation in vitro

**Fig. 2:**
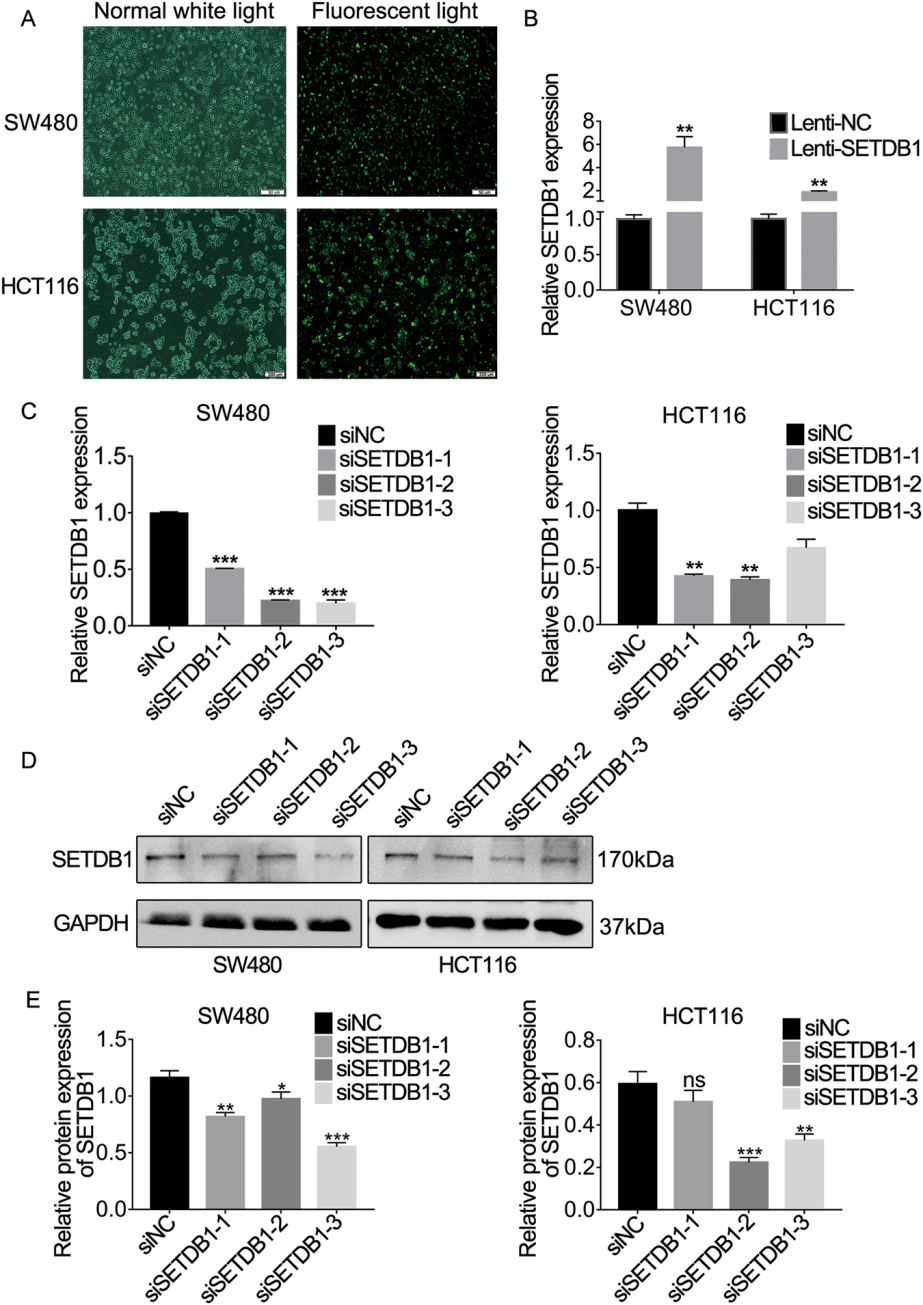
siRNA was selected for silencing SETDB1 or lentivirus overexpressing SETDB1 in CRC cells. **a** At 24 h post transfection, fluorescence photographs of CRC cells transfected with FAM-labeled siRNA using lipo2000. SW480: Scale bar, 50 µm; HCT116: Scale bar, 200 µm. **b** qRT-PCR analysis of SETDB1 expression following the treatment of SW480 and HCT116 cells with lenti-SETDB1 or the lenti-NC. **c**, **d** SETDB1 mRNA and protein expression in SW480 and HCT116 after transfected with siSETDB1-1, 2, 3 or the negative control. **e** Gray analysis of WB results by image J software. Error bars indicate mean ± SD. *P < 0.05, **P < 0.01, ***P < 0.001, ns means no significance.

**Fig. 3:**
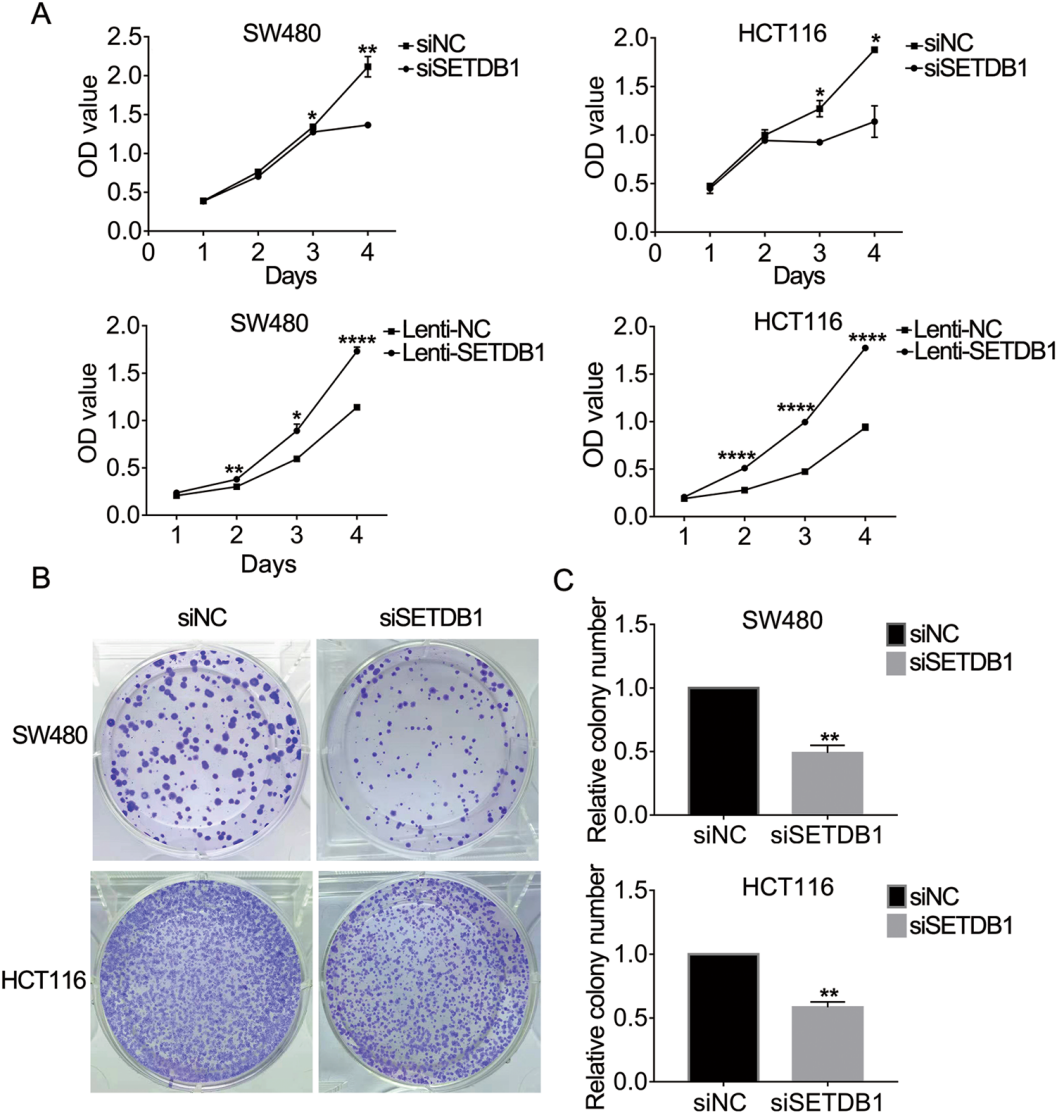
SETDB1 promotes CRC cells proliferation in vitro. **a** CCK8 assay was performed to detect the cell viability of siSETDB1 or Lenti-SETDB1 transfected CRC cells. **b** Colony assay was performed to analyze the proliferation of SW480 and HCT116 cells following treatment with siSETDB1 or siNC. **c** The number of cell clones was counted and analyzed. All the data come from three independent experiments. Error bars indicate mean ± SD. *P < 0.05, **P < 0.01, ***P < 0.001.

Since the expression of SETDB1 was increased in CRC, we investigated the biological function of SETDB1. We silenced or overexpressed the expression of SETDB1 by siRNA or lentivirus. We preliminarily evaluated the siRNA transfection efficiency in CRC cells through fluorescence image 24 h after transfection (Fig. 2a), qRT-PCR and WB were performed to verify the silencing or overexpression efficiency of SETDB1 (Fig. 2b–d). According to the results of qRT-PCR and protein gray analysis (Fig. 2c, e), we selected the most effective siRNA for further study, which is siSETDB1-3 and siSETDB1-2 in SW480 and HCT116 cells, respectively. In addition, we recorded them as siSETDB1 in the following experiments. To detect whether SETDB1 regulates the proliferation of CRC cells, we performed CCK8 and colony formation assay. The results showed that growth capacity and colony number of SW480 and HCT116 cells dramatically decreased following silence of SETDB1 (Fig. 3–c). Overexpressed SETDB1 promotes CRC cells proliferation (Fig. 3a). As a result, SETDB1 was associated with CRC cell proliferation.

### Silence of SETDB1 promotes G1-phase arrest and causes apoptosis of CRC cells

**Fig. 4:**
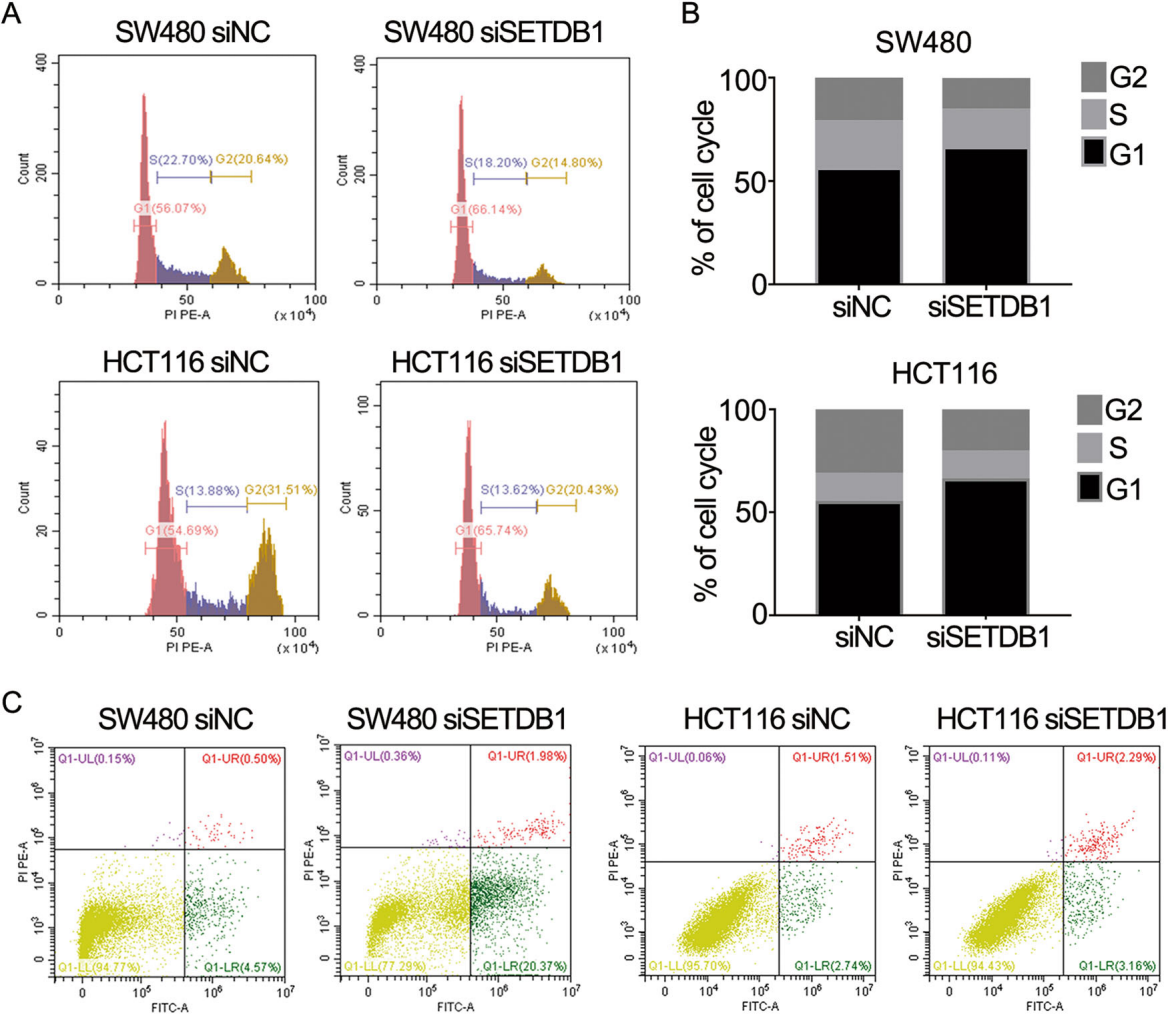
SETDB1 silencing promotes G1-phase arrest and induces apoptosis of CRC cells. **a** Changes of CRC cells cycle after transfection with siSETDB1 or siNC. **b** Cell cycle distribution of CRC cells transfected with siSETDB1 was represented in a histogram. **c** Flow cytometry was used to measure the apoptosis rate of SW480 and HCT116 cells transfected with siSETDB1.

To assess whether the effect of SETDB1 on CRC cells proliferation was mediated by cell cycle progression change, flow cytometry was performed to measure the cell cycle of CRC cells. As shown in (Fig. 4a, b), downregulation of SETDB1 significantly increased the G1-phase ratio and reduced the S-phase ratio. In addition, the apoptosis level of CRC cells was assayed by flow cytometry, which also reflected the growth activity of CRC cells. The results exhibited that knockdown of SETDB1 notably increased the apoptosis level of CRC cells (Fig. 4c). These studies implicate that SETDB1 has an important effect on CRC cells through cell cycle and apoptosis.

### SETDB1 promotes the migration and invasion of CRC cells

**Fig. 5:**
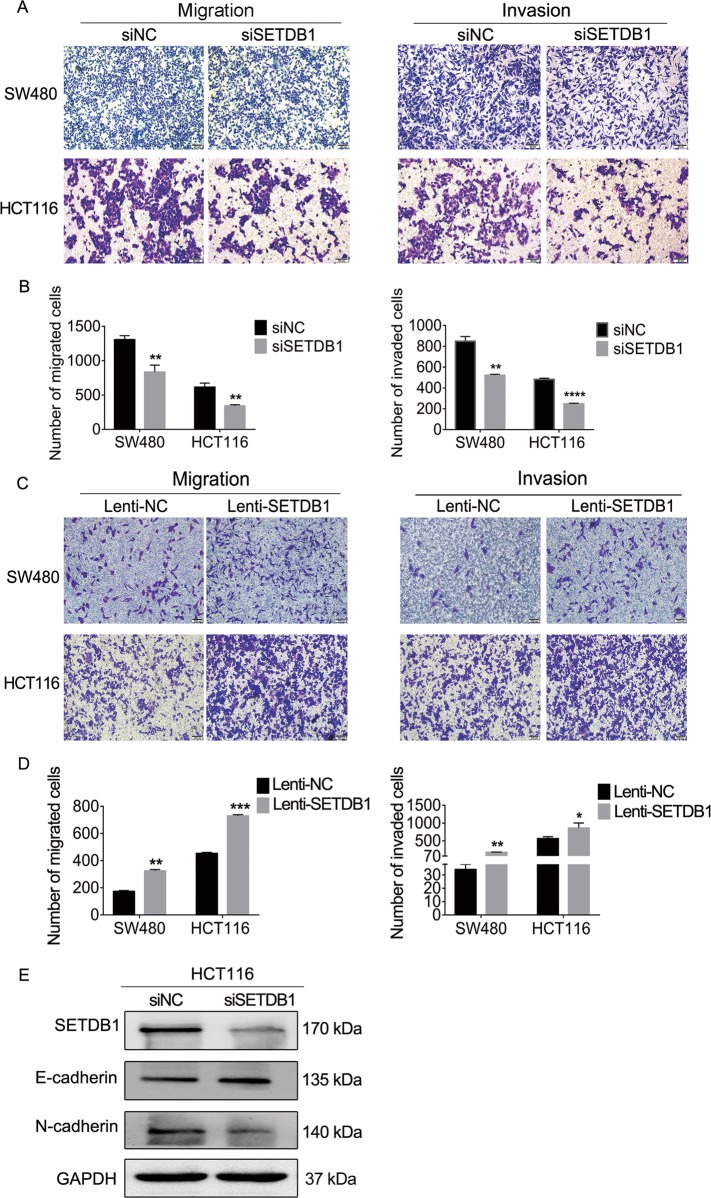
SETDB1 regulates migration/invasion and EMT progression in CRC cells. **a** Migration and invasion assays were performed after SETDB1 knockdown. SW480 migration: Scale bar, 20 µm. HCT116 migration and invasion: Scale bar, 50 µm. **b** The numbers of migrated/invaded cells were counted in three random fields by microscope. **c** Cell migration and invasion assays after transfected with Lenti-SETDB1 in SW480 and HCT116 cells. Scale bar, 200 µm. **d** Migrated and invaded cells of overexpressing SETDB1 were counted. **e** Protein of EMT markers in siSETDB1-knockdown HCT116 cells. The data are means from three independent experiments ±SD. *P < 0.05, **P < 0.01, ***P < 0.001.

SETDB1 has been reported to be involved in the regulation of EMT in breast cancer^26,39,40^. However, there is no report about the relationship between SETDB1 and EMT in CRC. In our experiment, we proved that SETDB1 did influence the EMT process in CRC cells. Cell migration and invasion assay indicated that the numbers of migrated and invaded cells were reduced in siSETDB1 group compared to the control, respectively (Fig. 5a, b). Ectopic expression of SETDB1 promoted CRC cells migration and invasion (Fig. 5c, d). In addition, the epithelial characteristic protein E-cadherin was increased, while the expression of mesenchymal characteristic protein N-cadherin was decreased after silencing SETDB1 in HCT116 (Fig. 5e). Therefore, our results indicate that SETDB1 may play an important role in the process of EMT in CRC cell lines.

### SETDB1 epigenetically silences p21 transcription

**Fig. 6:**
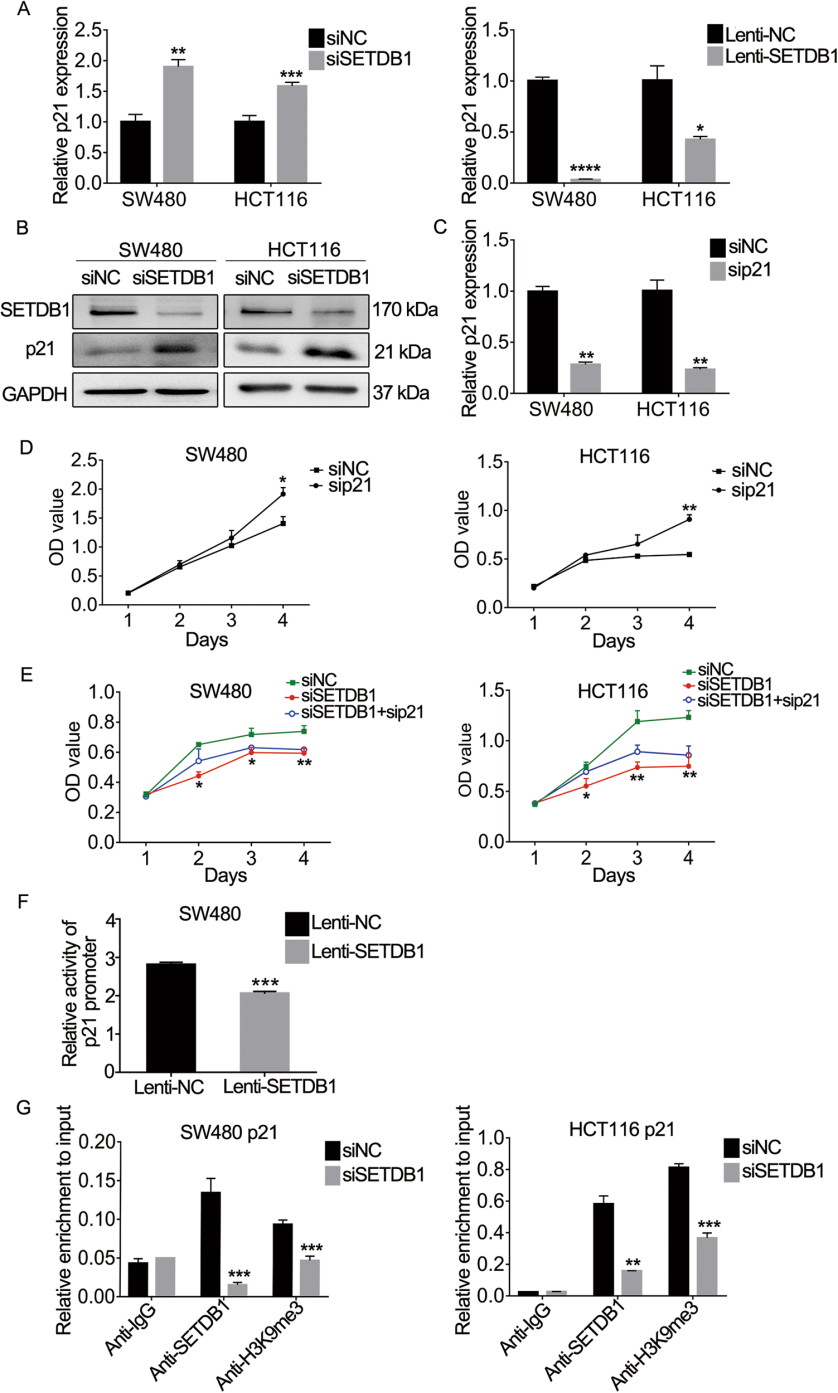
SETDB1 epigenetically silences p21 expression. **a** mRNA expression of p21 following the treatment with siSETDB1 or LV-SETDB1 in SW480 and HCT116 cells. **b** Protein expression levels of SETDB1 in SW480 and HCT116 cells after transfected with siSETDB1. **c** p21 expression in SW480 and HCT116 after treated with sip21. **d** Cell proliferation was analyzed by CCK8 in SW480 and HCT116 cells transfected with sip21 or siNC. **e** CCK8 assay was performed to monitor the proliferation of siSETDB1 and sip21 co-transfected CRC cells. **f** Detection of p21 promoter activity after overexpression SETDB1 in SW480 cells by dual-luciferase reporter assay. **g** ChIP-PCR was performed to validate the occupancy of SETDB1 and H3K9me3 modification at the p21 promoter in SW480 and HCT116 cells transfected with siSETDB1 or siNC, IgG as a negative control. the results represent the mean ± SD. All experiments were performed in triplicate. *P < 0.05, **P < 0.01, ***P < 0.001.

Although SETDB1 has been reported to play a critical role in CRC, the underlying mechanism remain unclear. To further explore the molecular mechanism, we used the UNIHI online database to predict the protein interactions and defined the target gene regulated by SETDB1^41,42^. We identified a tumor suppressive gene p21, which involves in cell cycle progression. According to the previous report and our data, we hypothesized that SETDB1 plays a role via regulating the expression of p21. To verify this hypothesis, we performed qRT-PCR and WB in CRC cells transfected with siSETDB1 or Lenti-SETDB1. The results showed that the expression of p21 was significantly upregulated after transfecting with siSETDB1 in SW480 and HCT116 cells but markedly decreased following SETDB1 overexpression (Fig. 6a, b). The efficiency of silencing p21 expression was verified by qRT-PCR (Fig. 6c). CCK8 assay indicated that downregulation of p21 promoted CRC cell proliferation and cells viability co-transfected with siSETDB1 and sip21 were higher than those transfected with siSETDB1 or sip21 alone (Fig. 6d, e). These results suggested that p21 could inhibit CRC cell proliferation and may be a downstream target gene of SETDB1. In order to clarify how SETDB1 regulates the expression of p21, we performed the dual-luciferase reporter assay in SW480 cells, as the result showed that overexpression of SETDB1 could inhibit the activity of p21 promoter in SW480 (Fig. 6f). Furthermore, we carried out the ChIP experiment using the specific antibodies of SETDB1 and H3K9me3 to enrich the DNA fragments combined with them and designing the specific primer of p21 promoter to detect the enrichment of p21, we found that the p21 promoter sequences enriched by the two antibodies were significantly reduced in the siSETDB1 group compared with the siNC group (Fig. 6g). Combined with the results of dual-luciferase reporter assay, we speculated that SETDB1 could bind to p21 promoter and regulate its activity, and silencing SETDB1 could reduce the enrichment of H3K9me3 on p21 promoter. In conclusion, SETDB1 contributes CRC development at least partly by epigenetically silencing p21 expression.

### Silencing of SETDB1 inhibits CRC tumorigenesis in vivo

**Fig. 7:**
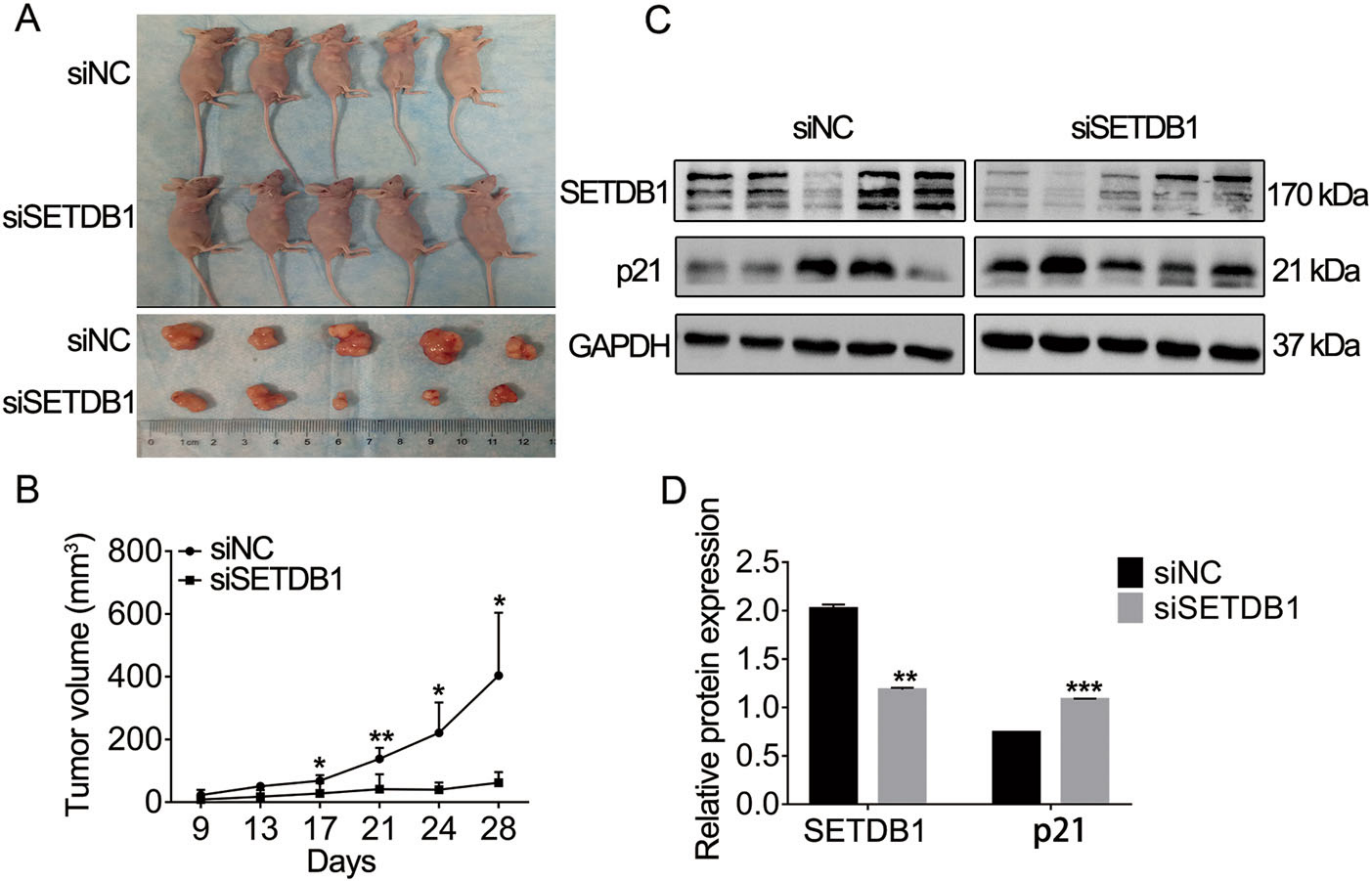
Silencing SETDB1 inhibits the CRC cells proliferation in vivo. **a** Tumor images of SETDB1-knockdown and control nude mice (*n* = 5/group). **b** Tumor volumes of the SETDB1-knockdown and control groups. The tumors were measured every 3 days. **c** SETDB1 and p21 protein expression in xenograft tumors transfected with siSETDB1 or siNC. **d** Grayscale analysis of WB results by image J software. The results show as the mean ± SD. *P < 0.05, **P < 0.01, ***P < 0.001.

Then, using a xenograft model in nude mice, we investigate the function of SETDB1 on the CRC in vivo. BALB/c nude mice were injected subcutaneously with HCT116 cells. When subcutaneous tumors were observed 9 days later, siSETDB1 or siNC were injected intratumorally every three days and tumors volume were measured. All mice were sacrificed 28 days after HCT116 cells injection. The results exhibited that tumors size in the siSETDB1 group were much smaller than that in the siNC group (Fig. 7a). Similarly, the tumor growth rate of siSETDB1 was slower compared with control group (Fig. 7b). WB experiment results showed that the expression of SETDB1 was reduced in siSETDB1 group, and p21 expression was upregulated in the siSETDB1 group compared with control (Fig. 7c, d). Taken together, our results suggested that silencing of SETDB1 in vivo could inhibit the proliferation of CRC cells and upregulate the expression of the tumor suppressive gene p21.

## Discussion

Recent years, epigenetics attracted more attention and its effects in the diseases have been studied intensively, particularly in carcinogenesis. Some epigenetic markers have been included in medical guidelines^43^. Histone methylation modification is mediated by HMTs and histone demethylases and plays critical role in gene expression regulation. In general, H3K27me3 and H3K9me3 lead to gene repression, whereas H3K4me3, H3K79me3, and H3K36me3 are associated with active transcription^44^. SETDB1 is a lysine 9-specific methyltransferase, specifically trimethylating H3K9^18,45^. SETDB1 can promote the development of tumors by inducing H3K9me3 modification at promoters of some genes and has been demonstrated abnormally expressed in various human cancer conditions^46^. In hepatocellular carcinoma, SETDB1 is amplified in tumor tissues and regulates the proliferation of liver cancer cells through methylation of p53^47^. In lung cancer, the expression of SETDB1 is upregulated in cancer tissues and cooperates with SMAD2/3 to inhibit lung cancer metastasis^48^. In addition, recent studies have found that SETDB1 can trigger AKT K64 methylation to activate AKT kinase activity, leading to cancer^49,50^. In addition, a previous study showed that SETDB1 promotes CRC progression via binding to the promoter of TP53^27^. More recently, Yu et al. reported that SETDB1 promotes CRC proliferation through the STAT1-CCND1/CDK6 axis^28^. Using UALCAN, an online cancer database and specimens of CRC patients, we found the expression of SETDB1 was upregulated in CRC. In addition, our data suggested that SETDB1 significantly influenced CRC cells proliferation, apoptosis, migration, and invasion in vitro. These results are consistent to previous studies and suggest that SETDB1 contributes to the pathogenesis of various cancers and plays an oncogenic role in CRC.

In current study, we also discovered that the classic tumor suppressor genes p21 is a target gene of SETDB1. p21 is involved in cell cycle regulation, which has been reported as a critical molecule for inhibiting cell proliferation in CRC cells^51–54^. Previous reports have showed that increased DNA methyltransferase 1 (DNMT1) in Ppara^∆IE^ mice could promote carcinogenesis of CRC via repressing the transcription of p21 through DNA methylation^55^. Long noncoding RNA CRNDE promotes the proliferation of CRC cells partly through epigenetically silencing of DUSP5 and p21 expression by binding with EZH2 and induce H3K27me3 modification at the promoter of these genes^36^. LncRNA HOXA-AS2 promotes the progression of CRC via repressing the expression of p21 and KLF2 by binding with EZH2 and LSD1, which both regulate the histone methylation^37^. Therefore, p21 acts as a tumor suppressor gene in CRC, and histone methylation plays an important role in regulating its expression. In our study, we verified that SETDB1 could regulate the promoter activity of p21 in CRC cells through the dual-luciferase reporter assay. Moreover, the ChIP experiment further proved that SETDB1 could regulate the enrichment of H3K9me3 on p21 promoter. Bao et al. reported that lncRNA-p21 prevents cell reprogramming via associating with SETDB1 and DNMT to maintain the heterochromatin state of the gene^56^, which implicated that the epigenetic regulation of p21 is one of the important mechanisms to contribute to the physiological and pathological function. Here, we reveal a new regulatory mechanism of SETDB1 and p21 in CRC.

Furthermore, we demonstrated that downregulation of SETDB1 could inhibit HCT116 cells proliferation in vivo. It suggests that SETDB1 may be a potential therapeutic target. Dysregulation of epigenetic markers has been extensively reported in tumors and inhibitors targeting these epigenetic enzymes have been developed^57^. For example, some HDAC inhibitors vorinostat, romidepsin, and belinostat have been approved for some T-cell lymphoma and panobinostat for MM. Other HDAC inhibitors such as resminostat, practinostat are in phase I and II clinical trials for the treatment of solid malignancies including hepatocellular and prostate cancer^58^. Sulforaphane is one of the most potent HDAC inhibitors that can inhibit proliferation and induce apoptosis of CRC cells^59^. In addition, an early report showed that more than 20 HMT enzymes have been reported to be associated with the occurrence of CRC and drugs targeting histone methylation have been demonstrated promising therapeutic effects in preclinical CRC treatment^60^. For example, EZH2 and SUV39H1 inhibitors have shown efficacy for CRC treatment in preclinical. EZH2 inhibitor GSK346 and chaetocin, a fungal metabolite inhibiting SUV39H1 which regulates the methylation of H3K9, could reduce migration of CRC cells^61,62^. In conclusion, the treatment targeting epigenetic modification is a promising clinical therapeutic strategy.

In summary, our study illustrated that SETDB1 was upregulated in CRC tissues and serve as an oncogene in CRC. The function role of SETDB1 on CRC cell proliferation and tumorigenesis is partly through epigenetic silencing of p21 expression. SETDB1 might become a promising target for the diagnosis and therapeutic strategy of CRC.

## Materials and methods

### Specimen collection

A total of 60 pairs primary tumor tissues and corresponding normal tissues were collected from CRC patients who received surgical treatment at Zhongnan Hospital of Wuhan University (Wuhan, China) between August 2017 and February 2018. All patients were diagnosed by original histopathological detection and none of them received preoperative adjuvant chemotherapy or radiotherapy. The patients with non‐curative resection, cancer recurrence, severe injury of vital organs, or a history of autoimmune diseases were excluded. Samples of the collected tissues were preserved in liquid nitrogen. Clinical case data of patients were also collected. The collection of clinical specimens was approved by the clinical research institution review committee and ethics review committee of the Zhongnan Hospital, and every patient was informed of their consent.

### Cell culture and transfection

Cell lines SW480 and HCT116 were purchased from the China Center for Type Culture Collection (Wuhan, China), and cells had been authenticated for STR profiling and tested for mycoplasma by the vendor. All the cells were cultured in Dulbecco’s modified Eagle’s medium (DMEM) (HyClone, USA) containing 10% fetal bovine serum (FBS) (HyClone, USA), 100 U/ml penicillin, and 100 mg/ml streptomycin (Genom, China), at 37 °C with 5% CO2. Purinicin-inducible GFP-tagged lentiviral SETDB1 (Lenti-SETDB1) were designed and synthesized by Shanghai Genechem (Shanghai, China). Cells infected with lentivirus were selected by 2 μg/ml of puromycin (Sigma, USA) to obtain stably infected cell lines. siSETDB1 and FAM-labeled siNC were purchased from Guangzhou RiboBio (Guangzhou, China), and transfected into cancer cell lines using Lipofectamine 2000 (lipo2000) (Invitrogen, USA). Transfection efficiency of siRNA with lipo2000 was preliminarily assessed by fluorescence microscope (Olympus U-RFL-T, Japan) 24 h after transfection and the silencing effect of siSETDB1 was further verified by qRT-PCR and WB experiments.

### RNA extraction and qRT-PCR

The total RNA in tissues and cells were extracted with Trizol reagent (Invitrogen, USA), and the reverse transcription was performed using TOYOBO ReverTra Ace kit (TOYOBO, Japan). mRNA expression was quantified using quantitative reverse transcription PCR (qRT-PCR) on Biorad CFX (Biorad, USA), and GAPDH were selected as a housekeeping gene. The primers were designed and synthesized by TSINGKE Biological Technology (Wuhan, China), primer sequences were as follows: GAPDHF-5′GGAGCGAGATCCCTCCAAAAT3′, R-5′GGCTGTTGTCATACTTCTCATGG3′, SETDB1F-5′GGTGGTCGCCAAATACAAA3′, R-5′ GGAAGCATAGCCATCATCAA3′, P21F-5′ TGCCGAAGTCAGTTCCTTGT3′, R5′CATTAGCGCATCACAGTCGC3′. The expression levels of mRNA were calculated using the comparative CT (2-∆∆CT), and all experiments were performed with three biological replicates.

### Protein extraction and western blotting

Total protein in CRC lines and subcutaneous tumors was extracted using RIPA lysis buffer (Boyotime, China) according to the reagent instructions. Western blotting was performed with the specific antibody, SETDB1(1:1000, Proteintech, 11231-1-AP), p21(1:1000, proteintech, 10355-1-AP), GAPDH (1:1000, Proteintech, 60004-1-Ig), E-cadherin (1:1000, Cell Signaling Technology, 3195 T), N-cadherin (1:1000, Cell Signaling Technology, 13116-T).

### CCK8 assay

Cell viability was detected using CCK8 kit (Dojindo Molecular Technologies, Japan). After transfection of HCT116 and SW480 cells with siSETDB1 and Lenti-SETDB1 for 48 h, 1000 cells per well were seeded in 96-wells plates with five replicate wells. CCK8 was added into the wells mixing with cell culture medium and incubate for 3 h. The absorbance at 450 nm was measured with a microplate reader (ELX-800; BioTek, USA), and the growth curve was drawn according to OD value.

### Migration and invasion assay

Migration and invasion assay were performed using the transwell chambers (Corning, USA). One hundred microliters matrigel matrix (Corning, USA) was added into upper chamber and incubated at 37 °C for 1 h for the invasion assay. Two hundred microliters cell suspension about 1 × 10^5^ were seeded in upper chambers with serum-free DMEM medium while the lower chamber was filled with 10% FBS. Cells were stained with crystal violet (sigma-Aldrich, USA) and photographed by microscope (Olympus, Japan).

### Apoptosis and cell cycle assay

Cell apoptosis assay was performed using Annexin V-FITC/PI apoptosis detection kit (BestBio, China), and cell cycle assay was performed using cell cycle and apoptosis detection kit (Boyotime, China). Cells were collected after 50 h transfecting siRNA, and all operations were performed according to the reagent instructions.

### Colony formation assay

After 24 h of siRNA transfection, 500 cells were seeded into a six-well culture plate and incubated at 37 °C with 5% CO2 and we performed the transfection again in these cells 4 days later, visible colonies could be observed at 9 days. The colonies were fixed with 4% paraformaldehyde and stained with crystal violet. Counting the number of colonies that made up of at least 50 cells.

### Tumor xenograft in nude mice

siRNAs were synthesized and purchased from Guangzhou RiboBio and chemically modified in the form of 2 ‘Ome + 5’ Chol (Guangzhou, China). Chemically modified siRNAs are very stable which could be used in vivo assays^63,64^. The sample size of animal experiment was determined as five nude mice in siSETDB1 group and siNC group and it was carried out randomly. We were not blinded for animal experiment. Two hundred microliters cell suspension (about 5 × 10^6^ HCT116 cells in PBS) were injected into female BALB/C nude mice at 4 weeks old, five mice per group. siRNA was injected into tumors when the subcutaneous tumor is visible at 9 days and we injected the siRNA into the tumors every 3 days to maintain its function. The tumor size was measured every 3 days using calipers. Mice were sacrificed at 28 days and the subcutaneous tumor was excised and preserved at −80°C. Animal experiments were approved by the Animal Ethics committee of Wuhan University.

### ChIP-PCR assay

CRC cells were fixed with 1% formaldehyde and incubated at room temperature for 10 min to make DNA-protein cross-links. Then glycine was added to stop the crosslinking and incubated at room temperature for 5 min. One milliliter cell lysis containing protease inhibitors (MCE, USA) was added to suspend cells and then cell lysates were sonicated using EPISONIC (USA) to get 200–300 bp of chromatin fragments. Immunoprecipitation was performed with SETDB1 (1:100, Proteintech, 11231-1-AP), H3K9me3 (1:50, CST, D4W1U) specific antibodies and IgG (1:100, CST, 2729S). The chromatin DNA was extracted using DNA purification kit (TIANGEN, China) and the specific primers of p21 promoter were used for PCR. The primer sequences were as following: p21F 5′-TGCATTGGGTAAATCCTTGCC-3′, R5′-AATGAGTTGGCACTCTCCAGG-3′.

### Dual-luciferase reporter assay

The high expression cell line of SETDB1 was constructed by infecting SW480 cells with lentivirus, and then the recombinant plasmid pGL4-basic-p21 was co-transfected with the control plasmid pRL-TK, 24 h after transfection, promoter activity was analyzed using a dual-luciferase assay kit (Promega, USA) according to the manufacturer’s instructions.

### Statistics analysis

In this study, all the experiments were performed in three times and data were exhibited as mean ± SD. The sample sizes for relevant experiment were determined by power analysis. When the variance between the two groups was similar, student’s *t* test was used to analysis data difference between two groups, if not the same, welch’s *t*-test was used, and chi-squared test was used to analysis clinical data. Statistics analysis was performed using SPSS 17.0 software (IBM, USA) and GraphPad prism 7.0 software (GraphPad software, USA). *p* < 0.05 was considered to be statistically significant.
